# When are Gallbladder Polyps Malignant?

**DOI:** 10.1155/2000/34201

**Published:** 2000-08

**Authors:** Dirk J. Gouma

**Affiliations:** Department of Surgery Academic Medical Center Meibergdreef 9 Amsterdam 1105 AZ The Netherlands


Gregory Van Stiegmann
Professor of Surgery
Head: GI Tumor and Endocrine Surgery
University of Colorado Health Sciences Centre
4200 East Ninth Avenue
Denver, Colorado 80262
USA
Fax: +303 315 5527

### References

D'Amico,, G. and Luca, A. (1997). Natural History: clinical-haemodynamic correlations: prediction of the risk of bleeding. *Baillieres Clin. Gastroenterol.*, **11**, 243–56.

Burroughs, A. K., D'Heygere, F. and McIntyre, N. (1986). Pitfalls in studies of prophylactic therapy for variceal bleeding in cirrhotics. *Hepatology*, **6**, 1407–13.

Lay, C.-S., Tsai, Y.-T., Teg, C.-Y., Shyu, W.-S., Guo, W.-S., Wu, K.-L. and Lo, K.-J. (1997). Is banding an acceptable treatment for varices that have not bled (prophylaxis)? *Hepatology*, **25**, 1346–50.

Shahi, H. M. and Sarin, S. K. (1998). Prevention of a first variceal bleed: an appraisal of current therapies. *Am. J. Gastroenterol.*, **12**, 2348–58.

Angelico, M., Carli, L., Piat, C., Gentile, S. and Capocaccia, L. (1997). Effects of isosorbide-5-mononitrate compared with propranolol on first bleeding and long-term survival in cirrhosis. *Gastroenterology*, **113**, 1632–39.

Garcia-Tsao, G., Groszman, R., Fisher, R. L., Conn, H. O., Atterbury, C. E. and Glickman, M. (1985). Portal pressure, presence of gastroesophageal varices and variceal bleeding. *Hepatology*, **5**, 419–24.

Pagliaro, L., D'Amico, G., Sorensen, T. I. *et al.* (1992). Prevention of first bleeding in cirrhosis: a meta-analysis of randomized trials of nonsurgical treatment. *Ann. Int. Med.*, **117**, 59–70.

The Veterans Affairs Cooperative Variceal Sclerotherapy Group. (1991). Prophylactic sclerotherapy for esophageal varices in alcoholic liver disease: a randomized, single blind, multicenter clinical trial. *N. Engl. J. Med.*, **324**, 1779–84.

Tait, I. S., Krige, J. and Terblanche, J. (1999). Endoscopic band ligation of esophageal varices. *Br. J. Surg.*, **86**, 437–46.

Laine, L. and Cook, D. (1995). Endoscopic ligation compared with sclerotherapy for treatment of esophageal variceal bleeding: a Meta-analysis. *Ann. Int. Med.*, **123**, 280–7.

Sarin, S. K., Guptan, R. C., Jain, A. K. and Sundaram, K. R. (1996). A randomized controlled trial of endoscopic variceal band ligation for primary prophylaxis of variceal bleeding. *Eur. J. Gastroenterol. Hepatol.*, **8**, 337–42.

Sarin, S. K., Lamba, G. S., Kumar, M., Misra, A. and Murthy, N. S. (1999). Comparison of endoscopic ligation and propranolol for prevention of variceal bleeding. *New. Engl. J. Med.*, **340**, 988–93.

Burroughs, A. K. and Patch, D. (1999). Primary prevention of bleeding from esophageal varices. *New. Eng. J. Med.*, **340**, 1033–5.

Poynard, T., Cales, P., Pasta, L. *et al.* (1991). Beta-adrenergic-antagonist drugs in the prevention of gastrointestinal bleeding in patients with cirrhosis and esophageal varices – an analysis of data and prognostic factors in 589 patients from four randomized clinical trials. *N. Engl. J. Med.*, **324**, 1532–8.

Lebrec, D., Bernuau, J., Rueff, B. and Benhamou, J. P. (1982). Gastrointestinal bleeding after abrupt cessation of propranolol administration in cirrhosis. *N. Engl. J. Med.*, **307**, 560.

Svoboda, P., Kantorova, I., Ochmann, J., Kozumplik, L. and Marsova, J. (1999). A prospective randomized controlled trial of sclerotherapy *vs.* ligation in the prophylactic treatment of high risk esophageal varices. *Surg. Endosc.*, **13**, 580–4.

De, B. K., Ghoshal, U., Das, T., Santra, A. and Biswas, P. K. (1999). Endoscopic variceal ligation for primary prophylaxis of oesophageal variceal bleed: preliminary report of a randomized controlled trial. *J. Gastroenterol. Hepatol.*, **14**, 220–4.

### When are Gallbladder Polyps Malignant?

### ABSTRACT

*Furukawa, H., Kosuge, T., Shimada, K., Yamamoto, J., Kanai, Y., Mukai, K., Iwata, R. and Ushio, K., Small Polypoid Lesions of the Gallbladder. Differential Diagnosis and Surgical Indications by Helical Computed Tomography. Arch Surg.,* **133**, *735–739.*

***Objectives:*** **To demonstrate the helical computed tomographic (CT) features of small polypoid lesions of the gallbladder and to establish a clinical strategy based on CT findings for the treatment of such lesions.**

**Design Validation cohort study.**

**Setting Tertiary care public hospital.**

**Patients Thirty-one patients with polypoid lesions of the gallbladder (≤3cm) underwent CT followed by resection.**

***Main Outcome Measure:*** **The detectability of the lesions on both unenhanced and enhanced CT and the configuration of the lesions on enhanced CT were prospectively evaluated in comparison with the histopathological findings.**

***Results:*** **Unenhanced CT detected 14 (45%) of the 31 lesions, whereas enhanced CT detected all of the lesions. The detection rates of the neoplastic**

**lesions (adenoma, adenocarcinoma, and metastatic tumor) and cholesterol polyps were 81% (13/6) and 7% (1/15), respectively (*P*<.001). Among the 20 lesions demonstrated as pedunculated, 6(30%) were neoplastic, whereas 10 (91%) of the 11 lesions demonstrated as sessile were neoplastic (*P*≤.001). When a lesion was demonstrated on unenhanced CT or its shape was sessile on enhanced CT, the case was diagnosed as a neoplastic lesion. The sensitivity, specificity, positive predictive value, negative predictive value, and overall accuracy of the CT diagnosis of the neoplastic lesions were 88% (14/16), 87% (13/15), 88% (14/16), 87% (13/15), and 87% (27/31), respectively.**
***Conclusion:*** **Computed tomography can differentiate neoplastic and nonneoplastic small polypoid lesions of the gallbladder and reliably identify the presence of neo-plastic lesions that should be resected.**

*Keywords:* Helical computed tomography, gallbladder carcinoma.

### PAPER DISCUSSION

The report of Furukawa *et al.*, is another study attempting to differentiate benign polypoid lesions of the gallbladder from neoplastic lesions or early gallbladder carcinoma by the use of non-invasive radiological techniques in order to establish a clinical strategy for treatment.

Thirty one consecutive patients who underwent resection for a polypoid lesion of the gallbladder were included in the study. Patients with small polyps (less than 5mm) and hyperechoic masses, thought to be a cholesterol polyp, were excluded and one should realize that false negative findings (missing a neoplastic lesion) can not be analysed for the entire cohort of patients with a "polypoid lesion".

The authors found that detectability of lesions is much higher (100%) on enhanced CT (EHCT) compared with unenhaced CT (UCT) (45%). The lesions on EHCT were classified as pedunculated 20/31 (65%) and sessile type 11/31 (35%). Most sessile lesions were malignant and cholesterol polyps are more frequently pedunculated. Most neoplastic lesions 13/16 were also identified with UCT, but UCT did not detect the malignant lesion in 3 patients (adenocarcinoma). Therefore, in an attempt to increase the accuracy, they used both techniques with the following criteria; when a lesion was found on UCT or its shape was sessile on EHCT, it was considered as a neoplastic lesion. This strategy increased the overall diagnostic accuracy to 87%.

In past years the size of a polypoid lesion (more than 10mm) was considered an important criterium to remove the gallbladder [1]. In the present study none of the lesions less than 10 mm was malignant and using this old strategy none of the malignant lesions would not have been operated. Cholecystectomy was also performed, however, in 10 patients with cholesterol polyps and two patients with adenoma. The introduction of this new above mentioned strategy might prevent these unnecessary cholecystectomies, however neoplastic lesions with curative options will be missed in 13%.

Shindoh recently suggested that unenhaced CT scan examination is effective for differentiation of benign and malignant lesions [2]. In the present study, however, only 81% of neoplastic lesions were found by UCT. Using these criteria (only UCT) there is doubt if the risk of missing a malignant lesion is acceptable.

It is not clear from both studies whether all patients who had neoplastic lesions not detected at UCT, did not have symptoms. This should be another argument to perform a cholecystectomy in these patients and probably detect the otherwise missed lesions and further decrease the chance of missing a malignancy. It has been shown previously that symptomatic patients with solitary polypoid lesions had a higher risk for developing gallbladder carcinoma [3].

Studies on the natural history of polypoid lesions are limited. Moriguchi reported in an observational study of 5 years in 109 patients that the size of the lesion did not change in 88% and that there was no correlation between the initial size (≤5mm; 6–9mm; ≥10mm) and the percentage with a change in diameter during follow-up nor a correlation with age and patient's sex [4]. The authors concluded that most poylpoid lesions are benign.

Other are not in agreement and suggest an underestimation of the risk for malignancy, probably because of the patient selection (only 2 single lesions ≥10mm) and a more aggressive approach is recommended [5,6]. Another argument for an aggressive approach is the relatively simple initial surgical treatment with minimal morbidity and mortality and on the other hand the dismal prognosis of gallbladder carcinoma [1,7].

It has been suggested that small lesion in patients without symptoms should not be removed, except for those patients with enlarging lesions or thickening of the gallbladder wall during follow-up by ultrasonography or CT. In the present study the number of small lesions was limited, but all proved to be benign. Recently, however, Shinkai *et al.*, has reported that most cholesterol polyps (97%) are less than 10mm, but the mean diameter of adenoma was 6±3.4mm. Six percent of neoplasms were observed among polyps less than 5mm. Shinkai suggested an aggressive approach regardless of size, in particular for single lesions [3]. One should also realize, although the risk is low, that adenomyomatosis has a malignant potential and in a few patients even carcinoma associated with cholesterolosis has been described [1,8,9].

Most studies have relatively small patient numbers. In addition the selection bias of patients, the lack of sufficient follow-up in nearly all studies with a non surgical treatment strategy and the different findings between studies, preclude a proposal for an evidence based treatment strategy for polypoid lesions of the gallbladder.

Regarding the above mentioned risks, in particular single polyps in symptomatic older patients, polyps larger than 10mm or polyps with an increase in size should be removed. This should also be performed for other polypoid lesions, if patients are not willing to undergo long term careful follow-up.

The use of enhanced CT, however, could prevent unnecessary cholecystectomy in a number of patients, but radiological evaluation during follow-up is indicated.

Another issue not addressed in this discussion is the appropriate approach how to remove the gallbladder, laparoscopically or with an open procedure. For small low risk lesions a laparoscopic approach could be accepted. CT might also be helpful to identify high risk patients for a milignancy. An open approach should be preferred in these patients to prevent port site metastasis.

Dirk J. Gouma
Department of Surgery
Academic Medical Center
MEIBERGDREEF 9
Amsterdam 1105 AZ
Holland

### *References*

Aldridge, M. C. and Bismuth, H. (1990). Gallbladder cancer: the polyp-cancer sequence. *Br. J. Surg.*, **77**, 363–364.

Shindoh, N. (1996). CT findings of the small polypoid lesions of the gallbladder (2cm or less PLG): differentiation between benign and malignant disease on unenhaced CT (Japanese) *Nippon Igaku Hoshasen Gakkai Zasshi – Nippon Acta Radiologica.*, **56**(3), 102–108.

Shinkai, H., Kimura, W. and Muto, T. (1998). Surgical indications for small polypoid lesions of the gallbladder. *Am. J. Surg.*, **175(2)**, 114–117.

Moriguch, H., Tazawa, J., Hayashi, Y., Takenawa, H., Nakayama, E., Marumo, F. and Sato, C. (1996). Natural history of polypoid lesions of the gall bladder. *Gut*, **39**, 860–862.

Kyriacou, E. (1997). Natural history of polypoid lesions of the gall bladder. *Gut*, **41**, 577–578.

Johnson, C. D. (1997). Polypoid lesions of the gall bladder. *Gut*, **41**, 578. (letter; comment).

Gouma, D. J. and Meyenfeldt, M. F. (1992). Prognostic factors for the survival time in gallbladder carcinoma. (Dutch). *Nederl Tijdschr Geneeskd.*, **136**, 225–229.

Aldridge, M. C., Gruffaz, F., Castaing, D. and Bismuth, H. (1991). Adenomyomastosis of the gallbladder, A premalignant lession? *Surgery*, **109**, 107–110.

Furukawa, H., Takayasu, K., Mukai, K. *et al.* (1995). CT evaluation of small polypoid lesions of the gallbladder. *Hepatogastroenterology*, **4**, 800–810.

